# Fatal Heat Stroke in a Schizophrenic Patient

**DOI:** 10.1155/2012/924328

**Published:** 2012-11-06

**Authors:** María Jesús Gómez Ramos, Francisco Miguel González Valverde, Carmen Sánchez Álvarez, Lisa Ortin Katnich, Francisco Pastor Quirante

**Affiliations:** ^1^Department of ICU, Reina Sofía General University Hospital, University of Murcia, 30.003 Murcia, Spain; ^2^Department of Surgery, Reina Sofía General University Hospital, University of Murcia, 30.003 Murcia, Spain; ^3^Department of Pathology, Reina Sofía General University Hospital, University of Murcia, 30.003 Murcia, Spain

## Abstract

*Objective*. The case of a patient who developed a fatal post-exertional heat stroke is reported. *Case Report*. A 20-year-old man with a history of morbid obesity, hypertension, and schizophrenia was admitted to our intensive care unit because of multiorgan failure due to severe heat stroke. He had been working under the sun. Treatment included aggressive body cooling but, in spite of the best supportive care, the patient succumbed in a few hours. We concluded that the adverse event was possibly associated with his obesity and the use of antipsychotics. Histological evaluation revealed lesions consistent with severe hyperthermia and shock. *Conclusions*. Heat stroke is an uncommon clinical entity characterized by systemic heat and loss of the body's normal mechanisms for dealing with heat stress, such as sweating and temperature control. When heat stroke is diagnosed early and supportive care begins promptly the prognosis is optimal but it becomes a life-threatening disease when treatment is delayed. Lack of physical acclimatization and the use of certain medications that interfere with salt and water balance can impair thermoregulation under conditions of high environmental temperature. Health professionals must be adequately prepared to prevent, recognise, and treat them urgently.

## 1. Introduction

Heat stroke (HS) is a potentially life-threatening disease characterized by an extreme elevation of core body temperature and neurologic disorders resulting in delirium, convulsion, or coma [1]. It is usually the result of exposure to high environmental temperature and strenuous exercise. Although treatable and preventable, a substantial number of people die from extreme heat in Europe each year.

The most frequent cause of death directly attributable to heat is HS but heat conditions are known to aggravate chronic disorders and leads to increased all-cause mortality, specially circulatory and respiratory mortality. So, the reported incidence of heat-related mortality is imprecise and grossly underdiagnosed in specific risk groups as the elderly, those with chronic medical diseases or morbid obesity, and the socially isolated [2]. 

## 2. Case Report

In late May 2008, a man aged 20 years was working in a field in Murcia (south-eastern Spain) for 4 hours in 30°C heat. He had a history of arterial hypertension, morbid obesity, and schizophrenia. His medications included risperidone 6 mg, biperiden hydrochloride, and enalapril 20 mg daily. At 4 pm he complained of dizziness and headache. After about 10 minutes he came running out in a highly agitated state and suddenly collapsed. The patient stayed under direct sunlight during approximately 2 hours until emergencies arrived and found him moaning and unresponsive (Glasgow Coma Scale 7: E2, V2, M3), with eye deviation, and breathless. His skin felt very hot and dry, blood pressure was 80/25 mmHg, pulse rate 176 bpm, and his axillary temperature—which underestimates core temperature—39.6°C. The patient was given intravenous fluids, intubated, rapidly cooled, and transported to our hospital, 50 minutes away.

On arrival to the ICU the patient was deeply sedated and still hypotensive (50/20 mm Hg). At this time his rectal temperature was 41.8°C. Electrocardiogram revealed sinus tachycardia with diffuse subendocardial lesion in ST; serum levels of myocardial markers were remarkably high and diffuse hypokinesis was observed on the echocardiogram. Chest X-ray was compatible with an early pulmonary edema. The cerebral CT scan did not reveal any pathology and, to exclude an infectious origin, a lumbar puncture was performed yielding normal cerebrospinal fluid.

Laboratory results revealed severe acidosis (pH 7.11, PCO2 70 mmHg, HCO3 17) and abnormal serum levels: creatinine (2.6 mg/dL) as well as blood urea nitrogen (BUN: 30 mg/dL) were elevated indicating the beginning of renal failure. Sodium was 138 mmol/L, potassium 4.6 mmol/L and chloride 115 mmol/L. Haematology analysis presented low platelet count (41.000) and severe alteration of coagulation levels (prothrombin time 13%, partial thromboplastin time >200 sec, INR 737, and fibrinogen 70). Hematocrit was 52.9%, hemoglobin 189 g/L, and leukocyte count 28400 (58% neutrophils and 12% band cells).

During the first hours, temperature control methods were continued with intravenous cold fluid infusion, vitamin K, sodium bicarbonate 1 molar, platelet, and fresh frozen plasma therapy. Conventional external cooling devices such as cooling blankets, cold compresses, and fans, warm water sprayed, and a gastric irrigation with cold water did not lead to any significant decrease in body temperature. 

One hour of intensive care later, core body temperature was 39°C. The patient started bleeding by venous access and gastrointestinal tube. Because of subsequent deterioration of the patient's condition and insufficient temperature control, use of vasopressors (noradrenaline) and hemodynamic monitoring with pulmonary artery catheter was required. Despite aggressive therapy, multiorgan dysfunction syndrome with anuria and disseminated intravascular coagulopathy developed and the patient died 9 hours after his access to ICU.

**Figure 1:**
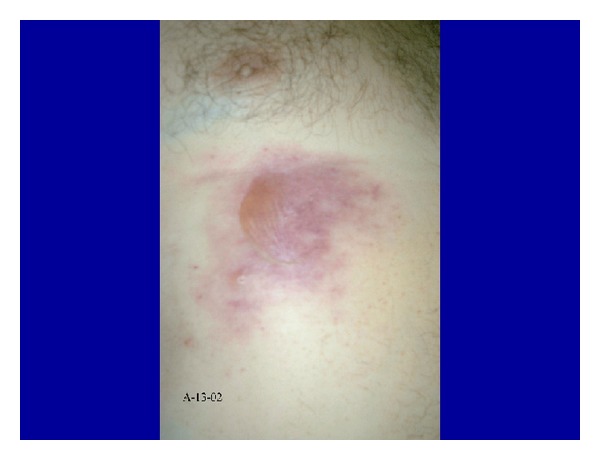
Superficial burn blisters with epidermal loss on non-exposure areas.

**Figure 2:**
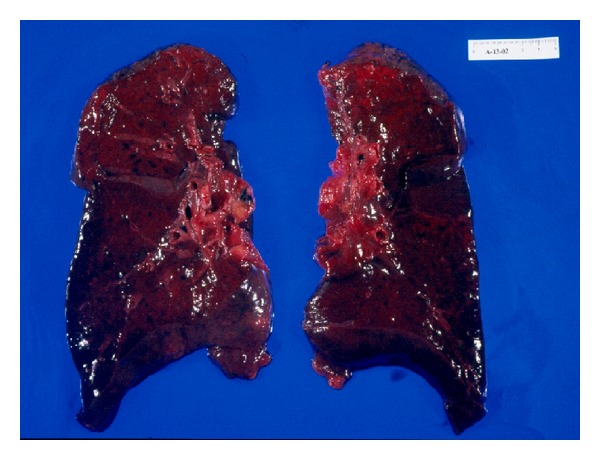
The lungs were heavily congested and hemorrhagic.

**Figure 3:**
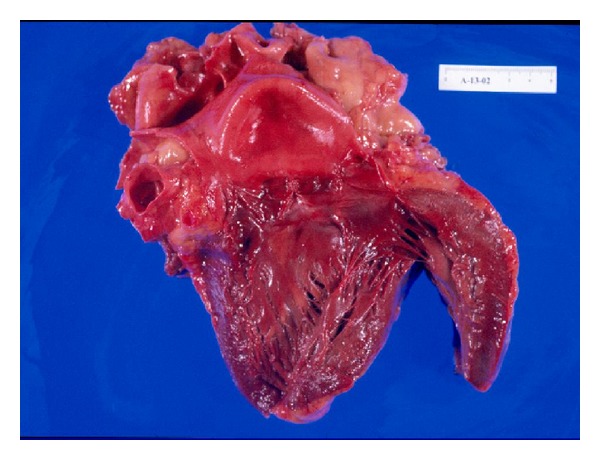
Heart specimen showing acute subendocardial-myocardial infarction.

In the autopsy, severe superficial skin burns with epidermal loss were observed on groin, neck, trunk, axillae, and other non-exposure areas (Figure 1). The lungs, the larynx, and the high respiratory airways were heavily congested and hemorrhagic (Figure 2). Histological study also revealed moderate brain edema, necrosis of the renal tubules, subendocardial necrosis (Figure 3), numerous petechial haemorrhages, and generalized visceral congestion. The underlying cause of death was heat stroke.

## 3. Discussion

The incidence of heat-related disorders is higher during the summer but it can also occur in moderate conditions, depending on environmental factors, age, and the use of several drugs (Table 1). The continuum of classic heat-related illnesses includes mild disease (heat edema, rash, cramps, syncope), heat exhaustion, and the most severe form, heat stroke. The last two situations are commonly treated in critical care units because of the highest risk of morbid-mortality. Clinically HS is distinguished from heat exhaustion by disturbances of the central nervous system, usually prolonged unconsciousness and coma often preceded by confusion, delirium, ataxia, or convulsions [3, 4].

Although HS has been classically documented as a medical condition, a universally accepted definition is lacking because its pathophysiology is not fully understood. It must be considered in anyone who presents with hyperthermia (exceeding 40°C) and altered mental status. Despite adequate hypothermia or other care-therapy, permanent neurological damage occurs in approximately 20% of patients and the mortality rate may be as high as 10 to 80% [1, 4–6].

Hyperpyrexia and neurologic dysfunction are necessary but not sufficient to diagnose HS. Associated clinical manifestations such as extreme fatigue and flu-like symptoms; hot dry skin or heavy perspiration; nausea; vomiting; diarrhea; disorientation; dizziness; uncoordinated movements; reddened face are frequently observed (Table 2). Clinical signs of dehydration and salt depletion are almost always present in the form of tachycardia, hypotension, and diaphoresis. Potential complications related to severe HS are acute renal failure, disseminated intravascular coagulation, rhabdomyolysis, acute respiratory distress syndrome, acid-base disorders, and electrolyte disturbances [4, 7, 8]. Above 42.4°C, thermal damage becomes critical, oxidative phosphorylation becomes uncoupled, proteins denature inducing changes in the membrane fluidity, and enzyme systems are affected [4]. HS resembles sepsis in many aspects, and endotoxemia and cytokines may be implicated in its pathogenesis [1, 9, 10].

Two forms of HS are recognized (Table 3): classic (no exertional), usually occurring in elderly persons with chronic illnesses, children, the obese, and those receiving medications such as diuretics, antipsychotics, antihypertensives, and antidepressants; exertional HS, more common in physically active individuals who develop a strenuous and exceptional exercise [3, 6, 8–10]. 

 In our case the patient was a morbidly obese subject that intook antipsychotics and antihypertensives, practising a strenuous activity at sunshine, and accompanied by inadequate fluid intake. Clinical manifestation was sudden and concurrent with exertional type but he had no sweating and hypoglycaemia, presenting several characteristics of the classic type. Certain drugs may induce or worsen heat-related illnesses. Drugs with anticholinergic effects can inhibit sweating and reduce heat elimination. Neuroleptics and tranquillizers, such as phenothiazines, have combined anticholinergic and central thermoregulatory effects. The set point of the temperature regulation centre can be elevated by the antidopaminergic effect of antipsychotics, such as phenothiazines and thioxanthenes [11–13].

The diagnosis of HS is suspected in the presence of a markedly elevated temperature and changes in mental status following heat exposure. Medical work-up include chest X-ray, electrocardiogram, and lab work with a complete blood count, electrolytes, BUN and creatinine, liver enzymes, creatine kinase, prothrombin time and partial thromboplastin time, arterial blood gasses, and urinalysis. The differential diagnosis includes hyperthyroid storm, pheochromocytoma, central nervous system injury, infection, anticholinergic poisoning, drug ingestion, and neuroleptic malignant syndrome [3]. 

In autopsy, edema of the brain, leptomeninges, petechial haemorrhages, and neuronal degeneration may be observed. The cardiovascular system may show right heart dilatation, pericardial effusion, edema, degeneration, and necrosis of myocardial fibres. Pulmonary infarction and high airways submucosal haemorrhages have also been described. The kidneys are enlarged with numerous petechial haemorrhages as well as the gastrointestinal submucosa. The liver may be congestive but structural injuries are infrequently found. Skin scalds with different depth degree on non-exposure areas and rigid and contracted muscles with necrosis of fibres are possible [14–16].

Assuming that HS is an acute life threatening emergency, the prognosis can be greatly improved if the symptoms are recognised early and emergency measures are instituted promptly. The prognosis is poorest when treatment is delayed >2 hours [10, 17, 18]. Early management should include the use of cold water or watersoaked towels, support of organ-system function, fluid resuscitation and electrolyte replacement if possible, and immediate transfer to a hospital. Aggressive cooling measures should be continued until the core temperature reaches 39°C. There is controversy regarding which cooling techniques are most effective. A combination of cold-water immersion and evaporative methods (spraying the body with atomised 15°C water and warmed air) is the best option for treatment [19, 20]. Other methods include ice packs placed over the axilla, groin, and neck. Disadvantages include peripheral vasoconstriction that retard heat loss; induced shivering, resulting in increased internal heat generation. If shivering does occur, medications as meperidine and diazepam can inhibit it; extreme discomfort to patients and medical attendants; difficulty in monitoring and resuscitating vital signs of patient [8, 20]. Internal methods for cooling as cold water irrigation to the stomach or rectum may be used but only in addition to external cooling methods [20]. Peritoneal lavage and cardiopulmonary bypass in severe cases have been advocated but their clinic efficacy is debated.

The use of pharmacological agents as antipyretics or steroids is not helpful in the treatment of HS. The hypothalamic setpoint is not elevated as it is in fever so that aspirin and acetaminophen are ineffective; furthermore aspirin is contraindicated for its effect on platelets and clotting [1, 4, 8]. Supportive cares are necessary and respiratory, neurologic, and cardiac status must be specially monitored [21]. In a comatose patient, a cuffed endotracheal tube should be placed to protect the airway as respiratory assistance is indicated. Invariably a venous access to replace fluids is required even if the patient is not hypotensive. Furthermore hemodynamic monitoring with pulmonary artery catheter may be indicated. This technique helps us for fluidotherapy management and vasopressors drugs if hypotension is not controlled well. Other aspects of monitoring include urine output measurement as indicator of acute renal failure.

Due to the risk and the prognosis, the best treatment is prevention. It requires awareness of risk factors and appropriate hydration. 

## 4. Conclusions

Heat stroke is a true medical emergency requiring immediate admission to an intensive care unit. Early recognition and management with aggressive measures to lower the body temperature with other supportive therapies can substantially reduce the mortality. Emergency departments and public health agencies should be adequately prepared to prevent, suspect, and manage heat-related illnesses, especially during heat waves. 

## Figures and Tables

**Table 1:** Conditons contributing to the risk of heat illness.

	Risk factors	Mechanism
Physical conditions	Prolonged exertion	
	Fever	
	Dehydration	

Medications	Drugs of abuse: amphetamines, heroine, cocaine, LSD, ethanol.	Increase endogenous heat production
	Anticholinergic: tricyclic antidepressants, antispasmodics and phenothiazides.	Disrupt hypothalamic function and reduce sweating
	Beta-adrenergic and Calcium channel blockers	Inhibit the compensatory increase in cardiac output
	Diuretics	Produce a relative state of dehydration that affects central thermoregulation and sweating
	Others: antiparkinsonian agents, antihistamines	

Chronic illness	Cardiac conditions	
	Cystic fibrosis	
	Extensive skin disease	
	Hyperthyroidism	
	Psychiatric conditions	

Older age		

**Table 2:** 

Characteristic	Classic	Exertional
Health condition	Predisposing factors	Healthy
Age	Elderly	Younger
Conditions	High environmental temp	Occurs sporadically
Sweating	Usually absent	Present
Activity	Sedentary	Strenuous
Disseminated intravascular coagulation	Mild	Marked
Acute renal failure	<5% patients	25–30% patients
Lactic acidosis	Rare	Common
Hyperuricemia	Moderate	Severe
Hypocalcemia	Rare	Common
Hypoglycaemia	Rare	Common
Hypokalemia	Rare	Common
Rhabdomyolysis	Rare	Common
CPK	Mildly elevated	Marked elevated
Mechanism	Poor dissipation of environmental heat	Excessive endogenous heat production

Information from [3, 8].

**Table 3:** Heat illness.

	Edema	Cramps	Tetany	Syncope	Exhaustion	Stroke
Symptoms	Minimal clinical significance but important interstitial fluid accumulation	Cramps	Cramps, carpopedal spasm, and perioral and distal paresthesias	Nausea, sighing, yawning, restlessness, and orthostatic syncope	Flulike	Flulike
Central nervous system symptoms	NO	NO	NO	NO	NO	Present
Temperature	<41°C	<41°C	<41°C	<41°C	<41°C	>41°C
Sweating	Present	Present	Present	Present	Present	Absent
Mechanism	Unacclimatisation, peripheral vasodilatation	Unacclimatisation and negative sodium balance	Unacclimatisation and severe negative sodium balance	Unacclimatisation, dehydration and inadequate cardiac output	Excess sweating in a hot humid environment causing volume depletion	Heat production exceeds dissipation
Treatment	Periodic exercise, elevation of the legs, or diuretic medication	Oral sodium replacement	Oral or parenteral sodium replacement	Placing supine position, and replacing water deficit	Cool area to rest, placing supine position and replacing water deficit	Emergency treatment

## Abbreviations

HS: Heat stroke
pm: Post meridiem
bpm: Beats per minute.
